# Comparison of efficacy and safety between simultaneous integrated boost intensity-modulated radiotherapy and conventional intensity-modulated radiotherapy in locally advanced non-small-cell lung cancer: a retrospective study

**DOI:** 10.1186/s13014-019-1259-3

**Published:** 2019-06-13

**Authors:** Daquan Wang, Nan Bi, Tao Zhang, Zongmei Zhou, Zefen Xiao, Jun Liang, Dongfu Chen, Zhouguang Hui, Jima Lv, Xiaozhen Wang, Xin Wang, Lei Deng, Wenqing Wang, Jingbo Wang, Chunyu Wang, Xiaotong Lu, Kunpeng Xu, Linfang Wu, Wenji Xue, Qinfu Feng, Luhua Wang

**Affiliations:** Department of Radiation Oncology, National Cancer Center/ National Clinical Research Center for Cancer/Cancer Hospital, Chinese Academy of Medical Sciences and Peking Union Medical College, Beijing, China

**Keywords:** Non-small cell lung cancer, Simultaneous integrated boost, Intensity-modulated radiotherapy, Survival, Toxicity

## Abstract

**Background:**

Consistent results are lacking as regards the comparative effectiveness of simultaneous integrated boost intensity-modulated radiotherapy (SIB-IMRT) versus conventional intensity-modulated radiotherapy in patients with locally advanced non-small-cell lung cancer (LA-NSCLC). Therefore, we conducted a retrospective analysis to demonstrate the role of SIB-IMRT for patients.

**Methods:**

Patients who had histologically confirmed NSCLC, stage III disease and received thoracic IMRT between 2014 and 2016 were retrospectively reviewed. The survival, toxicities and dose to organs at risk (OAR) were compared among patients irradiated with different techniques. The SIB-IMRT plans were designed to deliver 45–59.4Gy (median: 50.4Gy) to PTV while simultaneously delivering 50-70Gy (median: 59.92Gy) to PGTV. As for conventional IMRT plans, a total dose of 50-70Gy (median: 60Gy) was delivered to PTV.

**Results:**

426 patients with stage III NSCLC were eligible for analysis, including 128 with SIB-IMRT and 298 with conventional IMRT. The SIB-IMRT group had more stage IIIB disease (69.5% vs. 53%, *P* = 0.002), larger planning treatment volumes (median: 504 ml vs. 402 ml, P<0.001), and a larger planning treatment volume/volume of lung ratio (median, 0.18 vs. 0.12, P<0.001). The median OS of the SIB-IMRT and conventional IMRT groups were 34.5 and 31.7 months, with the 2-year rate of 60.4 and 59%, respectively (*P* = 0.797). No difference in PFS, LRFS or DMFS was observed between the two techniques. Patients treated with SIB-IMRT got similar lung and esophageal toxicities versus those with conventional IMRT.

**Conclusions:**

SIB-IMRT may be an effective and safe option for patients with locally advanced NSCLC, especially for those with large mass or wide lymph node metastasis.

## Background

Approximately 30% of patients with non-small cell lung cancer (NSCLC) belong to the locally advanced stage [1], and definitive thoracic radiotherapy concurrently with chemotherapy is the standard care [2] [3]. However, in clinic, certain patients with large mass, extensive lymph node metastasis, or poor lung function, often failed to tolerate definitive radiotherapy because of high risk of treatment-related toxicities. Radiation-induced pneumonitis (RP) is the most important dose-limiting toxicity. The incidence of symptomatic RP was reported to be about 20–30% in NSCLC patients treated with concurrent chemoradiotherapy [4, 5]. It might result in a decreased treatment intensification, such as interruption of radiotherapy or low tolerance of chemotherapy.

Intensity-modulated radiation technique (IMRT) has been proven to improve tumor control and to reduce lung toxicities and lower cardiac doses [5, 6]. Currently, the radiation dose delivered to gross tumor volume (GTV) and clinical target volume (CTV) are generally same. In the trial of RTOG 0617, a homogeneous dose of 74 Gy to both gross and subclinical diseases did not improve OS. On the contrary, an increased death risk by 38% was observed, which might due to increased radiation-induced toxicities [7].

In the modern area, there is a growing interest in the delivery of IMRT-based simultaneously integrated boost (SIB). This approach could simultaneously confer a radical dose to the gross tumor while a relatively lower dose to the subclinical disease, which consequently might improve treatment tolerance for patients with complex conditions such as large mass, extensive lymph node metastasis, or poor lung function. Although previous study reported that a conventionally fractionated dose of 50Gy may be sufficient to eradicate subclinical disease [8], the clinical effectiveness of SIB-IMRT for LA-NSCLC remains to be confirmed, due to the respiration introduces complexity and the potential failures of the treatment margin.

In the present study, SIB-IMRT-based lung sparing technique was used to confer radical radiation to the gross tumor volume, while limiting lung irradiation dose. We aimed to compare the efficacy and toxicity profile between SIB-IMRT and conventional IMRT.

## Methods

### Patients

**Fig. 1:**
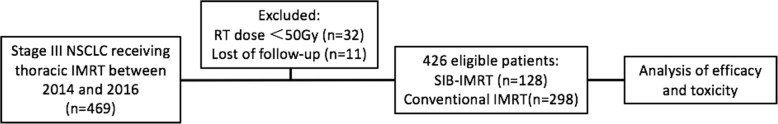
The procedure of data analysis

This was a retrospective study. Patients with histologically proven stage III NSCLC (American Joint Committee on Cancer, 7th edition) and receiving thoracic IMRT at our center between January 2014 and December 2016 were included. The procedure of data analysis was shown in Fig. 1. The inclusion criteria were histologically/cytologically confirmed NSCLC, older than age 18 at the time of diagnosis, stage III disease, Karnofsky performance status (KPS) score ≥ 70, and receiving thoracic IMRT. The enrolled patients had to have radiotherapy as a definitive form of treatment. Patients with radiation dose <50Gy, prior thoracic radiotherapy or surgery, or other coexisting primary tumors were excluded. The study was approved by the local institutional review board.

### Treatment strategy

**Fig. 2:**
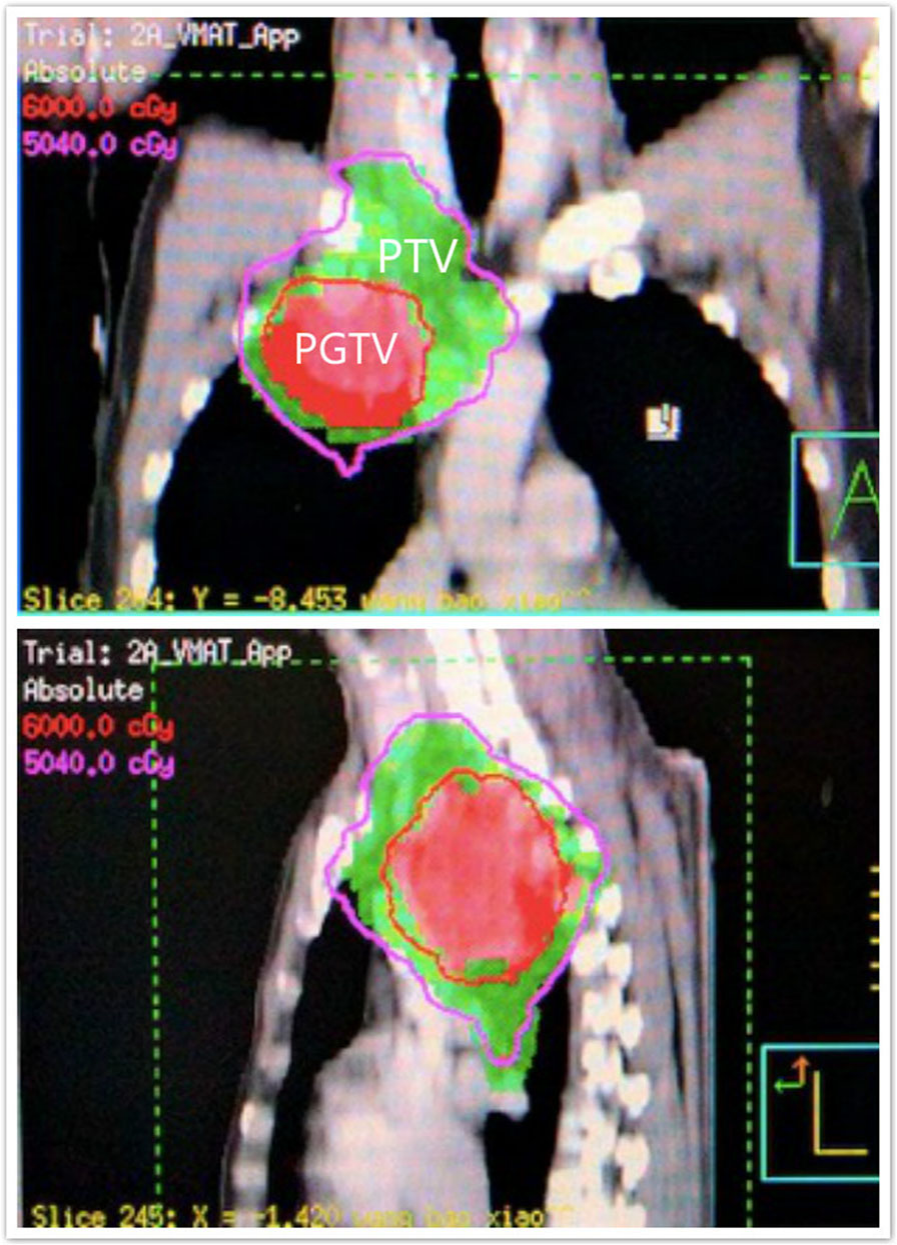
Dose distributions of IMRT plan with SIB technique. (PGTV: 59.92Gy/2.14Gy/28f, PTV 50.4Gy/1.8Gy/28f)

All the patients underwent computed tomograph (CT)-based simulation with 5-mm slice thickness. A head-neck-shoulder mask or chest mask was used to immobilize patients in the supine position during simulation. The scanned area extended from the laryngeal prominence to the bottom of the L2 vertebral body. Four-dimensional simulation was used for selected patients with peripheral or lower lobe lesions. The scanned images were transferred to a three-dimensional (3D) planning system (Philips, Pinnacle 9.0; Netherlands) (Fig. 2).

All of the patients underwent intensity-modulated radiotherapy (IMRT) [4]. The gross tumor volume (GTV) included the primary disease as well as any involved regional lymph nodes, which were defined as those with a short-axis diameter of at least 1 cm on the CT scan or with a short-axis diameter of less than 1 cm but with high fluorodeoxyglucose (FDG) uptake on the Positron emission tomography (PET)-CT scan. The clinical target volume (CTV) was created by expanding the GTV by 0.6–0.8 cm, as well as ipsilateral hilum and mediastinal nodal stations involved. The planning target volume (PTV) was generated by a uniform 0.5 cm expansion around the CTV. For patients treated with SIB-IMRT, the planning gross tumor volume (PGTV) was generated by a uniform 0.5 cm expansion around the GTV.

Radiation therapy was delivered by 6MV X-rays from a linear accelerator (Elekta, Synergy, Sweden; Elekta, VersaHD, Sweden; Varian, Novalis, United States; Varian, Unique, United States). The cone-beam computed tomograph (CBCT) scans was used in most patients for positioning. It was repeated before each treatment in the first week, then performed once a week. Electronic portal imaging device (EPID) was used in a small proportion of patients and it was conducted once a week. For patients treated with conventional IMRT, the median prescribed dose was 60Gy/30f (range: 50.0–70.0Gy, in 25–35 fractions) (Median BED_10_ 72Gy, range: 60-84Gy) to PTV. As for patients with SIB-IMRT, the median prescribed dose was 59.92Gy/28f (range: 50.0–70.0Gy, in 25–33 fractions) (Median BED_10_ 72.74Gy, range: 60-84Gy) to PGTV, and 50.4Gy/28f (range, 45–59.4Gy, in 25–33 fractions) (Median BED_10_ 59.47Gy, range: 53.1–70.1Gy) to PTV. It should be noted that the PTV in the SIB group contain the PGTV. All the patients received concurrent or sequential platinum-based doublet chemotherapy. The mean dose to the lungs (MLD) should optimally be ≤17Gy and not exceed 20Gy; the lung volumes minus GTV receiving more than 20Gy (V20) and 30Gy (V30) were limited to less than 30 and 20%, respectively. The heart volumes receiving more than 30Gy (V30) and 40Gy (V40), were limited to less than 40 and 30%, respectively. The esophageal volume receiving more than 50Gy (V50) was limited to less than 50%. The maximal dose for spinal cord PRV (5 mm) was 45Gy.

### Evaluation and follow-up

Pre-treatment evaluation included full medical histories and physical examinations, laboratory investigations, such as complete blood cell counts (CBC) and chemistries, electrocardiographs (ECGs), and pulmonary function tests. The following examinations were performed to specify the TNM stage of patients: chest and abdominal CTs, brain MRI/CTs, bronchoscopies, and radionuclide bone scans. PET-CTs were recommended but not mandatory.

All patients underwent CBC and blood chemistry examinations once a week during the treatment period. After discharge from the hospital, the patients were followed up every 3 months from hospital medical records and/or by phone. The follow-up evaluations consisted of patient history, a physical examination, and a thoracic CT at intervals of 3 months for 2 years and then 6 to 12 months for 3 years, or earlier if clinically indicated. Other imaging examinations were obtained when recurrence was suspected.

### Definition of endpoints

The endpoints included overall survival (OS), progression-free survival (PFS), locoregional recurrence-free survival (LRFS), distant metastasis-free survival (DMFS) and treatment-related toxicity. OS was calculated from the date of initial treatment to death or last follow-up, and PFS was calculated as the time to progression, death from any cause, or the last follow-up. Locoregional failures were defined as recurrences of primary tumor or regional lymph node, and distant metastasis were defined as disease progression excluding locoregional failure. Patients without evidence of progression were censored at the date of last follow-up or death. The treatment response evaluation was based on Response Evaluation Criteria in Solid Tumors (RECIST) version 1.1 [9]. Treatment-related toxicities were evaluated with Common Terminology Criteria for Adverse Events (CTCAE)4.0 criteria.

### Statistical analysis

Survival time was estimated using the Kaplan-Meier method, and the diffidence was examined by the log-rank test. Hazard ratios (unadjusted and adjusted) were estimated using Cox proportional hazards model. This model with backward method was also performed to identify significant variables. Chi-square test was used for dichotomous data comparison between groups. Continuous variables were compared by using the Mann-Whitney U test. All tests were two-sided, and *p* ≤ .05 was considered statistically significant. All the analyses were done using the SPSS software package (version 22.0, SPSS, Inc.)

## Results

### Patient characteristics

A total of 426 patients with stage III NSCLC were eligible for analysis, including 128 with SIB-IMRT and 298 with conventional IMRT. Patient and treatment characteristics of the two groups are summarized in Table 1. The median age for the entire cohort was 62 (range: 25–88) years, and 46% underwent concurrent chemoradiotherapy. SIB-IMRT group had more stage IIIB disease (69.5% vs. 53%, *P* = 0.002), larger planning treatment volumes (median: 504 ml vs. 402 ml, P<0.001), and a larger planning treatment volume/volume of lung ratio (median, 0.18 vs. 0.12, P<0.001). No significant difference was observed between the two groups regarding the distribution of age, gender, smokers, KPS, weight loss, histological classification, percentage of PET-CT staging, treatment modality, and radiation dose towards the PTV/PGTV (Table 1).

**Table 1:** Comparision of characteristics of the patients between SIB-IMRT and conventional IMRT

Characteristics	SIB-IMRT group (*n* = 128)	Conventional IMRT group (*n* = 298)	*P* value
Age (years)			0.934
≤ 70	110 (85.9%)	257 (86.2%)	
> 70	18 (14.1%)	41 (13.8%)	
Gender			0.76
Male	101 (78.9%)	239 (80.2%)	
Female	27 (21.1%)	59 (19.8%)	
KPS			0.213
≥ 80	124 (96.9%)	280 (94%)	
< 80	4 (3.1%)	18 (6%)	
Smoking			0.214
No	37 (28.9%)	68 (22.8%)	
Yes	91 (71.1%)	230 (77.2%)	
PET staging			0.486
Yes	47 (36.7%)	99 (33.2%)	
No	81 (63.3%)	199 (66.8%)	
Pathology			0.251
SCC	74 (57.8%)	190 (63.8%)	
ADE	47 (36.7%)	86 (28.9%)	
NSCLC	7 (5.5%)	22 (7.4%)	
TNM stage			0.002*
IIIA	39 (30.5%)	140 (47%)	
IIIB	89 (69.5%)	158 (53%)	
T stage			0.009*
T1	11 (8.6%)	10 (3.4%)	
T2	50 (39.1%)	86 (28.9%)	
T3	31 (24.2%)	100 (33.6%)	
T4	36 (28.1%)	102 (34.2%)	
N stage			0.001*
N0	1 (0.8%)	14 (4.7%)	
N1	6 (4.7%)	33 (11.1%)	
N2	48 (37.5%)	147 (49.3%)	
N3	73 (57%)	104 (34.9%)	
Treatment modality			0.874
RT alone	11 (8.6%)	28 (9.4%)	
Sequential CRT	59 (46.1%)	141 (47.3%)	
Concurrent CRT	58 (45.3%)	129 (43.3%)	
Prescribed dose (Gy)			0.775
≥ 66	11 (8.6%)	18 (6%)	
59–66	98 (76.6%)	237 (79.5%)	
55–59	12 (9.4%)	25 (8.4%)	
50–55	7 (5.5%)	18 (6%)	
PTV volume (ml)	504 (128–960)	402 (51–890)	< 0.001*
PTV/lung volume ratio	0.18 (0.04–0.43)	0.12 (0.01–0.32)	< 0.001*

*KPS* Karnofsky performance score, *PET* Positron emission tomography, *TNM* Tumor-node-metastasis, *SCC* Squamous cell carcinoma, *ADE* Adenocarcinoma, *RT* Radiotherapy, *CRT* Chemoradiotherapy, *PTV* Planning target volume

*p<0.05 was considered significant

### Response and failure pattern

The overall response rate was 81% for SIB-IMRT and 76.8% for conventional IMRT(*P* = 0.404). Locoregional recurrence rates were similar between the two groups (51.6% vs. 44.6%, *P* = 0.306). More details about failure pattern were shown in Table 2.

**Table 2:** Comparison of failure patterns of patients between SIB-IMRT and conventional IMRT

	SIB-IMRT group (*n* = 128)	Conventional IMRT group (*n* = 298)	*P* value
locoregional recurrence	45 (35.2%)	102 (34.2%)	0.853
distant-metastasis	56 (43.8%)	127 (42.6%)	0.829
locoregional+distant recurrence	21 (16.4%)	31 (10.4%)	0.115

### Survival

**Fig. 3:**
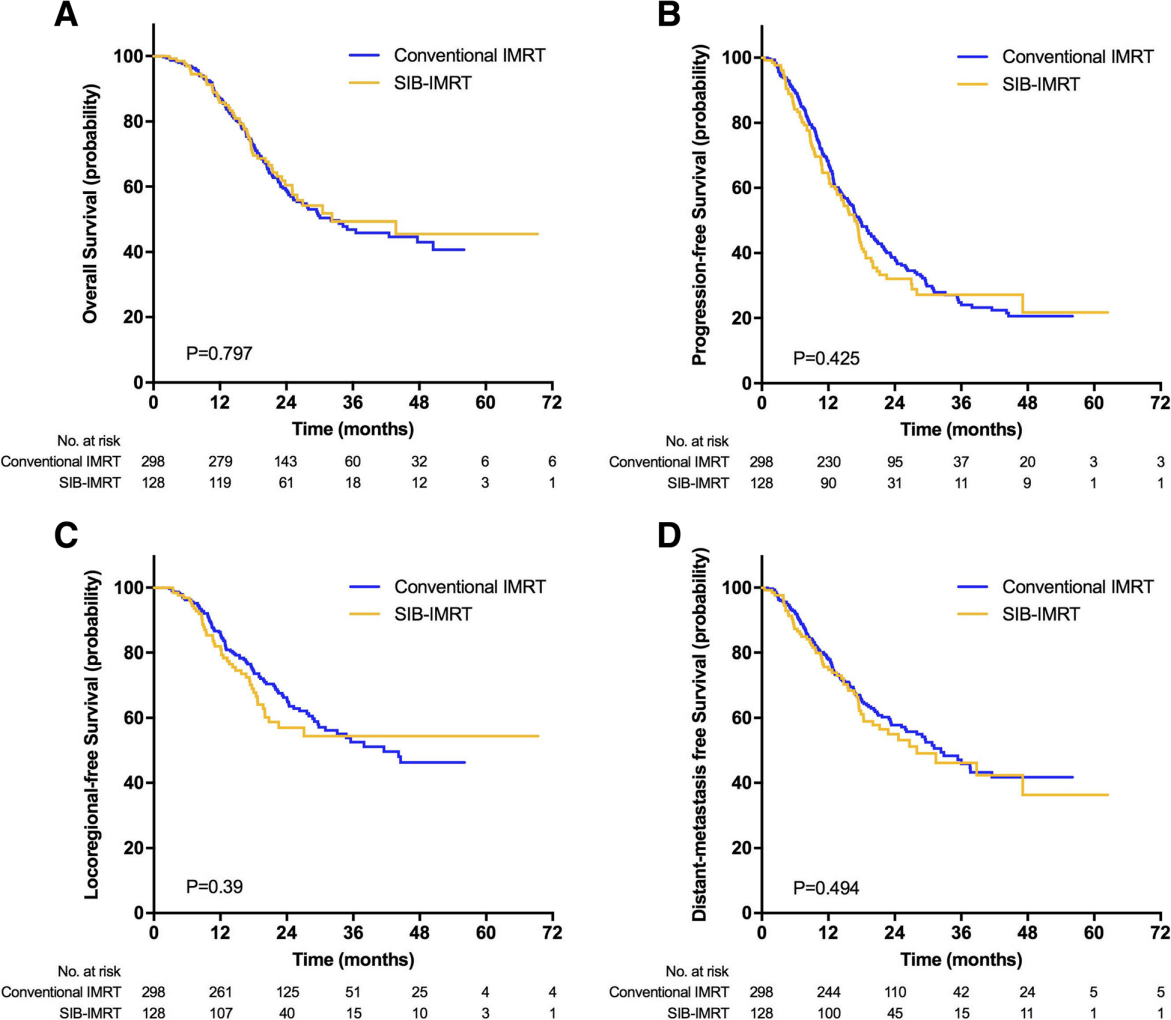
Comparison of survival between SIB-IMRT and conventional IMRT in patients. **a** Overall survival, **b** Progression-free survival, **c** Locoregional-recurrence free survival, **d** Distant-metastasis free survival

One hundred and eighty-two patients died (52 in the SIB-IMRT group; 132 in the conventional IMRT group), and 242 patients were still alive with a median follow-up time of 25 months (8-69 months) (SIB-IMRT group: 24 months; Conventional IMRT group: 26 months). The 1, 2, 3-year OS rate was 85.8, 60.4 and 49.4% for the SIB-IMRT group and 87.2, 59 and 46.9% for the conventional IMRT group. The difference regarding OS between the two arms was not statistically significant (*P* = 0.797, HR 0.959, 95%CI 0.695–1.322). The 1, 2, 3-year PFS rate was 64.7, 32.1 and 27.2% in the SIB-IMRT group and 68.3, 38.7 and 24.8% in the conventional group (*P* = 0.425, HR 1.11 95%CI 0.858–1.436). The median OS time (MST) were 34.5 and 31.7 months in the SIB-IMRT and conventional IMRT groups, with median PFS of 16.8 and 17.8 months, respectively. No significant difference was observed regarding LRFS (53% vs 45% at 3 years, *P* = 0.39) and DMFS (53% vs. 48% at 3 years, *P* = 0.494) between the two groups (Fig. 3).

The variables of age, gender, KPS, pathology, TNM stage, PET-staging, PTV, concurrent chemoradiotherapy, SIB-IMRT, and RT dose were included for multivariate analysis. The results showed that KPS (HR 0.463, 95%CI [0.269–0.796], *P* = 0.005), PTV volume (HR 1.627, 95%CI [1.184–2.237], *P* = 0.003) and concurrent chemoradiotherapy (HR 0.48, 95%CI [0.348–0.660], P<0.001) were independent factors affecting OS (Table 4).

### Dose and toxicities of OARs

Dose of OARs and treatment-related reactions were collected. Higher lung doses (V5, V20, MLD), Heart dose (mean dose, V30) and spinal cord dose (maximal) were observed in patients receiving SIB-IMRT (Table 3). While patients with conventional IMRT got higher maximal dose of esophageal (*P* = 0.021). No significant difference was observed in toxicities between the two groups, including radiation pneumonitis, esophagitis, hematologic toxicities and gastrointestinal reactions (Tables 4 and 5).

**Table 3:** Comparison of radiation dose to OARs of patients between SIB-IMRT and conventional IMRT

variables	SIB-IMRT	Conventional IMRT	*P* value
	median (range)	median (range)	
Radiation dose (Gy)	59.92 (50–70)	60 (50–70)	0.627
Lung V5 (%)	60.98 (28.63–89.39)	52.29 (22.67–87.4)	<0.001*
Lung V20 (%)	25.26 (12.89–29.76)	22.82 (5.96–30.58)	<0.001*
Lung V30 (%)	17.7 (7.14–22.74)	17.35 (2.16–24.46)	0.113
Mean lung dose (Gy)	15.10 (7.11–18.81)	14.08 (6.72–18.96)	0.001*
Maximum esophageal dose (Gy)	63.28 (12.21–73.95)	64.86 (12.41–75.26)	0.021*
Mean esophageal dose (Gy)	26.78 (7.88–50.63)	25.91 (4.31–50.63)	0.202
Esophageal V40 (%)	36.75 (3.59–86.64)	35.47 (0–77.17)	0.063
Esophageal V50 (%)	26.28 (0.75–73.84)	28.25 (0–66.3)	0.571
Mean heart dose (Gy)	13.12 (8.53–31.62)	11.5 (29.6–31.69)	0.044*
Heart V30 (%)	15.51 (0.42–54.68)	12.92 (0–46.73)	0.025*
Heart V40 (%)	9.21 (0–31.48)	8.05 (0–29.86)	0.309
Maximum spinal cord dose (Gy)	38.44 (30.26–64.87)	37.77 (9.12–42.42)	0.002*
Maximum spinal cord PRV dose (Gy)	42.98 (4.43–68.44)	43.11 (9.97–50.49)	0..96

*V5* Volumes receiving more than 5Gy, *V20* Volumes receiving more than 20Gy, *V30* Volumes receiving more than 30Gy, *V40* Volumes receiving more than 40Gy, *V50* Volumes receiving more than 50Gy, *PRV* Planning organ at risk volume

*p<0.05 was considered significant

**Table 4:** Multivariate analysis for overall survival

Characteristics	HR 95% CI	*P* value
Age, years (> 70:≤70)		0.279
Gender (female: male)		0.575
KPS (≥80:<80)	0.463 (0.269–0.796)	0.005*
Pathology (ADE:SCC)		0.374
TNM stage (IIIB:IIIA)		0.803
PET staging (yes:no)		0.836
PTV (>535 cc:≤535 cc)	1.627 (1.184–2.237)	0.003*
Concurrent chemoradiotherapy (yes:no)	0.48 (0.348–0.660)	< 0.001*
SIB-IMRT (yes:no)		0.504
RT dose (>59Gy: ≤59Gy)		0.595

*KPS* Karnofsky performance score, *ADE* Adenocarcinoma, *SCC* Squamous cell carcinoma, *TNM* Tumor-node-metastasis, *PET* Positron emission tomography, *PTV* Planning target volume, *RT* Radiotherapy

*p<0.05 was considered significant

**Table 5:** Comparison of radiation-related toxicities of patients between SIB-IMRT and conventional IMRT

Adverse event	SIB-IMRT (*n* = 128)				Conventional IMRT (*n* = 298)				*P* value
	Grade 2	Grade 3	Grade 4	Grade 5	Grade 2	Grade 3	Grade 4	Grade 5	
Pneumonitis	22 (17.2%)	3 (2.3%)	0	5 (3.9%)	45 (15.1%)	3 (1%)	0	5 (1.7%)	0.337
Esophagitis	32 (25%)	4 (3.1%)	0	0	70 (23.5%)	4 (1.3%)	0	0	0.448
Skin toxicity	8 (6.3%)	1 (0.8%)	0	0	25 (8.4%)	2 (0.7%)	0	0	0.738
Leukopenia	30 (23.4%)	15 (11.7%)	2 (1.6%)	0	62 (20.8%)	28 (9.4%)	6 (2%)	0	0.868
Thrombocytopenia	7 (5.5%)	3 (2.3%)	1 (0.8%)	0	6 (2.0%)	3 (1%)	1 (0.3%)	0	0.262
Anemia	12 (9.4%)	0	0	0	18 (6%)	0	0	0	0.234
Vomiting	3 (2.3%)	1 (0.8%)	0	0	18 (6%)	0	0	0	0.069

## Discussion

Despite having more stage IIIB disease (65% vs 50%), larger volume of PTV (median, 504 ml vs 402 ml), and a larger PTV/lung volume ratio (median, 0.18 vs. 0.12), the group of SIB-IMRT achieved similar efficacy and toxicities compared with conventional IMRT. The results demonstrated that SIB-IMRT might be an effective and safe treatment option for patients with LA-NSCLC.

According to previous studies, the subclinical disease contained a lower tumor burden than gross tumor lesion, usually less than 10^8, and 45Gy–50Gy may effectively eradicate subclinical metastases [8, 10, 11]. The findings proved the theoretical feasibility of delivering a reduced dose to subclinical disease. Recently, several studies explored the application of SIB-IMRT in LA-NSCLC and the results were encouraging [12–14]. Although the dose delivered to CTV is reduced (median: 50.4–51.58Gy), the locoregional failures did not increase, with 2-year LRFS rates ranged from 62.5 to 66.1% [12, 13]. In the present study, the median dose delivered to PTV was much lower in the SIB-IMRT group (50.4Gy vs. 60Gy), but the 3-year LRFS rate was not compromising (53% vs. 45%, *P* = 0.39). This result suggested that a conventionally fractionated dose of 50Gy might be sufficient to achieve good control in subclinical regions. A noteworthy feature of SIB-IMRT is that the technique can obtain highly conformal dose distribution with sharp dose gradients. Therefore, CBCT is needed to ensure the accurate tumor localization and dose delivery [15]. Considering the costs and time involved, we conducted five daily CBCTs in the first week followed by weekly imaging. But additional CBCTs were performed when imaging changes were detected requiring potential treatment adjustment, in order to ensure the accuracy of radical radiotherapy.

In our study, a total of 128 patients received SIB-IMRT, with PGTV median dose of 59.92Gy (BED_10_ = 72.74Gy). The results showed that the one, two, three-year OS rate was 85.8, 60.4 and 49.4%, respectively. The outcome made an improvement to OS compared with previous literature [12–14]. This was probably due to a higher proportion of patients receiving CCRT in our study than in other studies (45% vs. 27.6% & 35.5%). Meanwhile, the multivariate analysis indicated that CCRT was an independent factor predicting favorable OS. Thus standard CCRT should be applied for patients treated with SIB-IMRT, in order to achieve better survival.

Since the radiation dose delivered to subclinical areas was reduced, patients with SIB-IMRT are supposed to have better OAR spring. Xia et al. [16] demonstrated that plan-SIB resulted in better OAR spring (including lung, heart, esophageal and spinal cord) than plan-routine in NSCLC. However, our dosimetric data did not present a reduction in OAR doses as expected. Patients with SIB-IMRT got higher lung dose (V5, V20, MLD), heart dose (mean dose, V30) and spinal cord dose (maximal). It may be due to the larger volume of PTV for SIB-IMRT compared with conventional IMRT (median: 504 ml vs 402 ml, *P* < 0.001=. We choose SIB-IMRT to cover larger target volumes, for purpose of obtaining acceptable normal tissue doses. With respect to treatment-related toxicity, grade ≥ 2 pneumonitis for the two groups were 23.4 and 17.8% (*P* = 0.337), respectively, which was consistent with previous reports [5, 17]. As for esophageal toxicity, we observed no significant difference between RT techniques (grade ≥ 2: 28.1% vs. 24.8%, *P* = 0.448). The present results indicate that it is a safe option to receive SIB-IMRT, and patients with large target volume have good tolerance to definitive radiotherapy with the technique.

Although the trial of RTOG 0617 failed to achieve the anticipated result, much interest remains in dose escalation. A retrospective analysis utilizing National Cancer Data Base enrolled 33,566 patients with stage III NSCLC treated by thoracic radiation, and the results showed that patients with dose of ≥66Gy had increased OS than those with dose of 59.4-60Gy (median: 21.1 vs 18.8 months) [18]. Dose-escalation is still a feasible tool to improve outcome if the toxicity can be well controlled [19]. SIB-IMRT seems to be a useful technique for dose-escalation, in which the increased dose was only delivered to GTV/PGTV [20]. A phase II trial conducted by Kong et al. [21] demonstrated that the dose-escalation using PET/CT-guided adaptive SIB-IMRT could achieve favorable local-regional control. Meanwhile, a retrospective study with small samples indicated that higher doses (>66Gy) to PGTV was associated with lower risk of distant metastasis [14]. In our study, the group of SIB-IMRT had larger target volume, and we supposed that most of them could not benefit from dose-escalation because of high risk of treatment-related toxicity. Therefore, only eleven patients received higher dose (≥66Gy) to PGTV. But the outcome was encouraging since only two patients (18.2%) had local-regional recurrence during follow-up. Further studies with larger samples are warranted to demonstrate the role of SIB-IMRT in dose-escalation for LA-NSCLC.

Because of the inherent flaws of a retrospective study, there are some limitations in the present study. Firstly, the imbalance of tumor size between the two arms may offset the benefit from SIB-IMRT. Secondly, the radiation dose and the chemotherapy regimens of the patients were not uniform. Last, because the health care system has not warranted coverage of PET for oncologic use, only 34.2% of the patients performed PET-CT before treatment in our study. But all the patients received comprehensive examinations to ensure the accuracy of TNM staging, including chest and abdominal CTs, brain MRI/CTs, bronchoscopies, and radionuclide bone scans.

## Conclusions

In conclusion, this retrospective single institution study showed that SIB-IMRT might be an effective and safe option for patients with locally advanced NSCLC, especially for those with high risk of treatment-related toxicities. It offered patients with large tumor volume the chance to receive a more intense treatment. The value of dose-escalation using SIB technique is desirable. Future prospective clinical trials are warranted to confirm the efficacy of SIB-IMRT in NSCLC patients.

## Abbreviations

CT: Computed tomograph
CTV: Clinical target volume
DMFS: Distant metastasis-free survival
GTV: Gross tumor volume
LRFS: Locoregional recurrence-free survival
MLD: Mean lung dose
NSCLC: Non-small cell lung cancer
OS: Overall survival
PFS: Progression-free survival
PGTV: Planning gross tumor volume
PTV: Planning target volume
SIB-IMRT: Simultaneous integrated boost intensity-modulated radiotherapy
